# Role of Expectation and Working Memory Constraints in Hindi Comprehension: An Eye-tracking Corpus Analysis

**DOI:** 10.16910/jemr.10.2.4

**Published:** 2017-04-04

**Authors:** Arpit Agrawal, Sumeet Agarwal, Samar Husain

**Affiliations:** Indian Institute of Technology Delhi, India

**Keywords:** sentence comprehension, surprisal, working memory constraints, incremental dependency parser, eye-tracking, Hindi comprehension

## Abstract

We used the Potsdam-Allahabad Hindi eye-tracking corpus to investigate the role of wordlevel and sentence-level factors during sentence comprehension in Hindi. Extending previous work that used this eye-tracking data, we investigate the role of surprisal and retrieval cost metrics during sentence processing. While controlling for word-level predictors (word complexity, syllable length, unigram and bigram frequencies) as well as sentence-level predictors such as integration and storage costs, we find a significant effect of surprisal on first-pass reading times (higher surprisal value leads to increase in FPRT). Effect of retrieval cost was only found for a higher degree of parser parallelism. Interestingly, while surprisal has a significant effect on FPRT, storage cost (another predictionbased metric) does not. A significant effect of storage cost shows up only in total fixation time (TFT), thus indicating that these two measures perhaps capture different aspects of prediction. The study replicates previous findings that both prediction-based and memorybased metrics are required to account for processing patterns during sentence comprehension. The results also show that parser model assumptions are critical in order to draw generalizations about the utility of a metric (e.g. surprisal) across various phenomena in a language.

## Introduction

Eye movements have been successfully employed to uncover cognitive processes that subserve naturalistic reading. Researchers who have been studying eye movements have been able to give us very precise models of eye movements along with establishing the link between eye movements and the underlying cognitive processes [38, 39, 8, 47].

An eye-tracking corpus typically comprises of naturalistic text with eye movement information of all the words that make up the text. Eye-tracking corpora have been used extensively in the area of reading research to model eye movement control in English and German [40, 10, 25, 24, 41]. For example, using the Potsdam Sentence Corpus, Kliegl et al. [25] showed a significant effect of word frequency, word predictability and word length on fixation durations in German. Their work also argued for a distributed nature of word processing [40]. The Potsdam Sentence Corpus consists of 144 German sentences with fixation duration data from 222 readers. The Dundee eye-tracking corpus [24] is another popular eye-tracking corpus for English. It contains eye-tracking data for 10 participants on 51,000 words of newspaper text in English.

While these corpora have played an important role in the reading research, they have also been used to investigate processing theories using naturalistic text in psycho-linguistics [11, 12, 36]. In particular they have been used to test both expectation-based (Hale, 2001; R. Levy, 2008)[18, 29] and working memory based theories [15, 31 ] of sentence processing. For example, Demberg and Keller [9], while investigating the Dundee eye-tracking corpus found that dependency locality theory (DLT) [14] successfully predicts reading times for nouns. They also found that an unlexicalized formulation of the surprisal metric [18] predicts reading times of arbitrary words in the corpus. Similarly, M. Boston, Hale, Kliegl, Patil, and Vasishth [5] used the Potsdam Sentence Corpus and found that surprisal models all fixation measures as well as regression probability in their data. Further, M. F. Boston, Hale, Vasishth, and Kliegl [6] used the same Potsdam Sentence Corpus to show that retrieval cost [31] is effective in modelling reading times only at a higher degree of parser parallelism. More recently, Frank, Monsalve, and Vigliocco [13] have constructed an eye-tracking corpora that is intended to serve as the gold standard for testing psycholinguistic theories for English. The data comprises of 361 independently interpretable sentences from a variety of genres; these sentences have different syntactic constructions and therefore the text is meant to be representative of English syntax.

While the relevance of eye movement has been known in the psychology and psycholinguistics literature for some time, it is only recently that eye movement data are being used in various natural language processing applications. For example, Barrett and Søgaard [1] used fixation patterns and fixation durations to automatically predict part-of-speech categories of words in a sentence. The key insight for this work is that reading research has demonstrated that fixation duration can correlate with word properties such as its category, e.g. function words are generally skipped while reading. Similar insights were used by them to also predict grammatical functions during parsing [2]. While the use of fixation duration for predicting part-of-speech tags and grammatical functions is quite intuitive, some researchers have been able to exploit eye-tracking-based features for as varied a task such as modelling translation difficulty [34], sentiment annotation complexity [22], sarcasm detection [35], and sentence complexity [42]. These works show that reading data is quite rich and has subtle eye movement patterns can be very useful in various applications.

Similar to the work on English and German [5, 9], in a recent work, Husain, Vasishth, and Srinivasan [21] used an eye-tracking corpus to investigate sentence processing in Hindi. They created the Potsdam-Allahabad Hindi Eye-tracking Corpus which contains eye movement data from 30 participants on 153 Hindi sentences. They used this corpus to show that during Hindi comprehension word-level predictors (syllable length, unigram and bigram frequency) affect first-pass reading times, regression path duration, total reading time, and outgoing saccade length. Longer words were associated with longer fixations and more frequent words with shorter fixations. They also used two high-level predictors of sentence comprehension difficulty, integration and storage cost[14, 15], and found a statistically significant effect on the ‘late’ eye-tracking measures.

The significant effect of storage cost in Husain et al. [21] is interesting because it is the first evidence in favor of this metric in a naturalistic text using the eye-tracking paradigm. Storage cost characterizes the effort required to maintain predictions of upcoming heads in a sentence. On the other hand, current evidence for predictive processing in head-final languages such as Japanese, German and Hindi support the predictions of the surprisal metric [18]. The surprisal metric is quite distinct from the storage cost. Surprisal is defined as the negative log probability of encountering a word given previous sentential context. In this study we investigate the contribution of these two expectation-based metrics, namely storage cost and surprisal, using the Hindi eye-tracking corpus. While Husain et al. [21] investigated the effect of integration cost in their study to capture working memory constraints during sentence comprehension, we also explore the effectiveness of an alternative working-memory cost – the cue-based retrieval cost [31].

Finally, we discuss the role of parser model assumptions, i.e. the parsing algorithm, feature set etc. on the model predictions. In order to do this we use the computed surprisal to model reading times of a self-paced reading experiment [20]. The reading time data in this SPR experiment is supported by predictions made by the surprisal metric. We therefore wanted to test if the experimental data can also be explained by the automatically computed surprisal values.

## Predictive processes in language comprehension

It has long been argued that human sentence processing is predictive in nature [32, 33, 28]. Recent work in sentence processing has conclusively established that prediction plays a critical role during sentence comprehension [26, 18, 23, 29], but see Huettig and Mani [19]. While the predictive nature of the processing system has been established, the exact nature of this system is still unclear

(1) Subject Relative: The reporter who sent the photographer to the editor hoped for a good

(2) Object Relative: The reporter who the photographer sent to the editor hoped for a good story

It has been proposed that a comprehensive theory should not only appeal to predictive processing but also be able to simultaneously account for working memory constraints. For example, in his eye-tracking study investigating processing difference in English object vs subject relative clauses such as (2) and (1), Staub [43] finds evidence for both expectation-based processing and locality constraints. But these opposing effects are seen at different regions in object relatives. While evidence for surprisal theory is seen at the first noun after the relative pronoun, locality-based effect (which have been argued to reflect working memory constraints) is seen as processing slowdown at the relative clause verb. This suggests that both types of processing accounts are needed in order to capture the experimental data. This idea has been further corroborated by many studies [30, 44, 20]. Husain et al. [21] also found the effect of working memory constraints (in terms of integration cost) as well as prediction (in terms of storage cost) in a Hindi eye-tracking corpus. However they did not test for surprisal which is an important metric that captures predictability. Given that both storage cost and surprisal quantify the predictive processes during comprehension and considering the fact that surprisal has considerable support from experimental work in various languages (including Hindi), we wanted to explore the relative contribution of these metrics in the Hindi eye movement data.

### Surprisal

Surprisal assumes that sentence processing is accomplished by using a probabilistic grammar. Using such a grammar the comprehender can expect certain structures based on the words that have been processed thus far. The number of such probable structures becomes less as more words are processed. Intuitively, surprisal increases when a parser is required to build some low probability structure. Following M. Boston et al. [5], we compute surprisal using prefix probabilities. For a given probabilistic grammar G, we define prefix probability at the *i^th^* word (αi) as the sum of probabilities of all partial parses (d) until the *i^th^* words. Surprisal at the *i^th^* word then is the logarithm of the ratio of prefix probability before and after seeing the word. Surprisal is always positive and in general, unbounded. In our computation, we only take the top k parses based on their likelihoods at each word to compute αi inEquation 1. 

**Figure:**
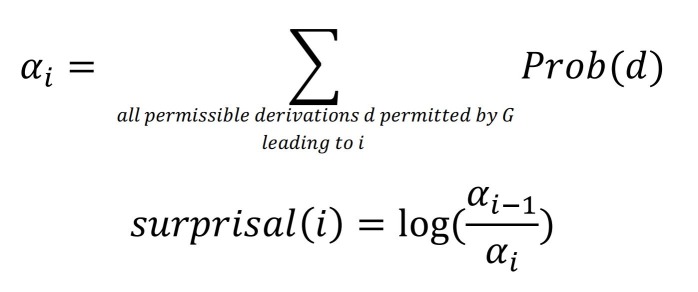


(3) dilli meediaa kaa makkaa-madinaa hai Delhi media GEN Mecca-Medina is ‘Delhi is the epicenter of the media (in India).

**Table 1:** Surprisal (k = 3) at different words for the sentence dilli meediaa kaa makkaa-madinaa hai – ‘Delhi is the epicenter of the media (in India).’

Word	Gloss	αi	Surprisal
dilli	Delhi	1	0.00000
meediaa	Media	0.99997	0.00003
kaa	GEN	0.9985	0.00148
makkaa-madinaa	Mecca-Medina	0.3134	1.15865
hai	Is	0.2713	0.14419

In sentence (3), the α (which is defined as the sum of probabilities of the top k parses) decreases as the sentence progresses, while the negative logarithm of the probability increases monotonically. Surprisal, thus is the difference of this increasing series. As mentioned previously, there is considerable cross-linguistic support for surprisal, both from eye-tracking data [9, 5, 6] as well as from experimental work in (among others) English [43], German [44, 30] and Hindi [20].

### Storage Costs

Storage cost (along with integration cost) is a metric proposed by Gibson [15] as part of Dependency Locality Theory (DLT). Storage Cost characterizes the processing load incurred as a result of maintaining predictions of upcoming heads in a sentence. To illustrate the diverging predictions of surprisal and storage cost, consider the following example:

(4) deepika ko shaam se abhay ne Deepika ACC evening INST Abhay ERG fona nahi kiyaa hai phone not did PRES ‘Abhay hasn’t called Deepika since evening’

The storage cost at deepika ko is 1 as a verb is predicted at this point in order for this sentence to end grammatically, this storage cost remains constant as new arguments are encountered before the verb. When the verb (fona kiyaa hai) is encountered the storage cost become 0. Surprisal will predict a processing cost at encountering abhay ne because encountering a noun phrase with an Ergative case at this position is rare (6% of the 175 Ergative-Accusative word order instances in the treebank had non-canonical word-order).

There is some evidence for storage cost from experimental data in English [14, 7] and from the eye-tracking data in Hindi [21].

## Methodology

Following, Husain et al. [21] we analyze the effect of certain word-level and sentence-level predictors on the eye-tracking measures. Below we list these dependent and independent variables. Finally, we discuss the parser details used to compute the surprisal values.

### Variables

Independent Variables/Predictors. All the predictor used in the Husain et al. [21] study are used in this study as well. Syllable length, word complexity, unigram and bigram frequencies are used as word-level predictors. Integration cost and storage cost were the sentence-level predictors. The details of the computation of these predictors can be seen in Husain et al. [21]. In addition we also use lexical surprisal for each word as a sentence-level predictor.

All predictors were scaled; each predictor vector (centered around its mean) was divided by its standard deviation.

Dependent Variables (Eye-tracking Measures). Again, following Husain et al. [21], we present analyses for one representative first-pass measure – first-pass reading time, and two representative measures that often show the effects of sentence comprehension difficulty – regression-path duration and total reading time [8, 47]. First Pass Reading Time/Gaze Duration on a word refers to the sum of the fixation durations on the word after it has been fixated after an incoming saccade from the left, until the word on the right is seen. Regression Path Duration/Go-Past Duration is the sum of all first-pass fixation durations on the word and all preceding words in the time period between the first fixation on the word and the first fixation on any word right of this word. Total Fixation Time is the sum of all fixations on a word.

In our study, storage cost was computed manually^1^. To estimate surprisal, we used an incremental transition-based parser. We implemented our own probabilistic incremental dependency parser in Python. The code for the parser is freely available online: https://github.com/samarhusain/IncrementalParser.

### Parsing Algorithm and Implementation Details

We use the incremental transition-based parsing algorithm (Arc-Eager) [37] to parse sentences in order to compute surprisal values for each word in a sentence. This is similar to the approach of M. F. Boston et al. [6]. However, unlike them we compute lexicalized surprisal. This is because an unlexicalized dependency parser for Hindi has very poor accuracy. We used the sentences in the Hindi-Urdu treebank (HUTB) [4] to train our parser. See Appendix for more details on the training data and parser accuracy.

A state in a transition-based parser comprises of (a) a stack, (b) a buffer, (c) a word position index, and (d) the partial parse tree. Arc-Eager is a transition-based parsing algorithm that allows four transitions to go from one state to the other. These states are LEFTARC, RIGHT-ARC, REDUCE and SHIFT. A transition may modify the stack, and/or the parse tree and/or may increment the index by at most one count. Not all transitions are allowed on all states. Before the parsing begins, the starting state consists of an empty stack, the buffer contains all the words of the sentence to be parsed, index is initialised to zero and since no structure has been formed yet, we have an empty parse tree. As part of the parsing process, transitions are applied incrementally till we reach a state where the parse tree is complete, or no transition is allowed on the state.

Our parser starts with the starting state mentioned above. In the first step, it creates a set of states that can be achieved by applying only one transition to the starting state. For example, we can use SHIFT to transfer the first word from the buffer on to the stack. In the second step, we create a set of states that can be achieved by applying only one transition to those states in the previous set, where the index is still 1. For example, given the first word on the stack, we can either apply LEFT-ARC, RIGHT-ARC or SHIFT. REDUCE is prohibited because the first word has not been assigned a head yet. We keep applying all possible transitions to each state, until all states have index 1. This is the set associated with index 1.

We now use this set and repeat the above procedure till we get a set that only has states with index 2. While applying these transitions, we might end up with some states on which no transitions are legal. We simply drop such states. Thus we keep creating these sets for each value of index starting from one.

As one would guess, the number of elements in the set increases exponentially with the index. Therefore to keep our algorithm tractable, we limit the size of the set of states corresponding to each index to utmost k most probable elements. We use a MaxEnt model to output probabilities of each transition we apply. The probability of a state is simply the product of the probabilities of all the transitions made to achieve that state.

The prefix probability corresponding to index i is the sum of probabilities of states corresponding to the index *i*. Surprisal at index *i* is computed as the log-ratio of prefix probability at index (*i*-1) and prefix probability at index *i*.

Here we briefly discuss the surprisal computations for each word in example (3). The surprisal values are shown in Table 1while maintaining k=3. When we see the first word dilli, there are four possible transitions according to the Arc-Eager algorithm. A REDUCE or LEFT-ARC operation is not possible at the first word hence we are left with only two possible partial parses. The maximum number of parses we can maintain is greater than that (since k=3), thus we do not discard any of the potential partial parses. As a result the probability at the first word is 1, and the surprisal is 0. As we move further in the sentence, we see the word meediaa. At this stage, each of the two partial parses from the previous word can give rise to multiple partial parses, the total number being six. Here the sum of the probabilities of all the six partial parses would be 1, but we only take the three most probable ones, the sum of whose probabilities is 0:99997, giving rise to a surprisal of 0.00003. Note that the surprisal value will be low when the probability of remaining k parses is higher. This happens when the probability mass is distributed less uniformly with some parses being much more probable than the others. In other words, surprisal is lower when the parser can figure out with a greater degree of certainty, which partial parse is the correct one. Note how in Table 1the post-position kaa has very little surprisal since post-positions routinely follow nouns. However, a proper noun such as makkaa-madinaa is not expected here (due to low frequency); this leads to a higher surprisal value.

## Analysis and Results

Linear mixed models were used for all statistical analyses. We use the R package 2 lme4 [3] for fitting linear mixed models^3^. In the lme4 models, cross varying intercepts and varying slopes for subjects and items was included. No intercept-slope correlations were estimated, as data of this size is usually insufficient to estimate these parameters with any accuracy.

Each word served as a region of interest. All data points recorded with 0 ms for these fixation measure (about 25% of the data) were removed, and the data analysis was done on log-transformed reading times to achieve approximate normality of residuals.

Tables 2, 3, 4 show the results for the three dependent measures. The result for first-pass reading time (Table 2) showed a significant effect of both word bigram frequency and syllable length; increase in syllable length leads to longer reading time, and increase in bigram frequency leads to faster reading time. In addition, we found a significant effect of surprisal^4^, increase in surprisal value leads to increase in the reading time. A significant effect of bigram, word length and integration cost was found for log regression path duration (Table 3). Increase in integration cost leads to increase in reading time; the significant effect of bigram frequency and word length are in the expected direction. Finally, barring surprisal, integration cost and word complexity, all other predictors are significant for log total fixation time (Table 4); these effects are in the expected directions. In particular, increase in storage cost leads to increase in reading time.

**Table 2:** Results of linear mixed-effects model on log first pass reading time.

	Estimate(b)	Std. Error	t value
Intercept	5.502	0.023	237.74
Word complexity	0.003	0.003	0.87
Word frequency	-0.0003	0.006	-0.04
Word bigramfrequency	-0.014	0.003	-4.00
Syllable length	0.112	0.011	9.95
Integration cost	0.004	0.004	1.00
Storage cost	0.003	0.006	0.50
Surprisal	0.013	0.004	2.88

**Table 3:** Results of linear mixed-effects model on log regression path duration.

	Estimate(b)	Std. Error	t value
Intercept	5.655	0.031	181.45
Word complexity	0.003	0.004	0.77
Word frequency	-0.005	0.007	-0.75
Word bigramfrequency	-0.023	0.003	-6.53
Syllable length	0.116	0.011	10.44
Integration cost	0.012	0.005	2.26
Storage cost	-0.011	0.007	-1.57
Surprisal	0.002	0.005	0.52

**Table 4:** Results of linear mixed-effects model on log regression path duration.

	Estimate(b)	Std. Error	t value
Intercept	5.619	0.030	181.32
Word complexity	0.005	0.002	1.97
Word frequency	-0.016	0.007	-2.24
Word bigramfrequency	-0.018	0.004	-4.41
Syllable length	0.131	0.010	12.06
Integration cost	0.001	0.004	0.39
Storage cost	0.019	0.006	2.80
Surprisal	0.005	0.004	1.14

## Discussion

The results shown in tables 2, 3, 4 are consistent with those reported in Husain et al. [21]. Like the previous study we find robust effect of word-level predictors, such as word frequency, bigram frequency, and word length. We also find a significant effect of sentence-level processing predictors, storage cost and integration cost in total fixation time and regression path duration respectively.

In this study we introduced a new sentence processing measure, surprisal, as a predictor to investigate different eye-tracking measures. The role of surprisal had not been explored by Husain et al. [21]. Our results show a significant effect of surprisal on log first pass reading time. Research on eye-tracking data in other languages such as English [9] and German [5]) have also found significant effect of surprisal. Our work supports this line of research. Interestingly, surprisal is a significant predictor in addition to bigram frequency. Since bigrams are known to capture local word predictability due to high collocation frequency, it can be argued that surprisal values in this study account for non-local syntactic predictability. Experimental studies on sentence processing in Hindi [45, 27, 20] have found evidence for predictive processing that can be explained through surprisal.

Further, our results also support previous research both using eye-tracking data [9, 5] as well as experimental data [43, 44, 29, 20] that have shown that both expectation-based metric as well as memory-constraint metric are required to explain processing in various languages such as English, German and Hindi. The results in this study show that surprisal (which captures expectation) as well as integration cost (which captures working-memory constraints) are independent predictors of reading time during naturalistic reading in Hindi. The significant effect of integration cost in our study goes contrary to certain proposals that have argued that head-directionality in a language determines locality vs anti-locality effects [30]. Interestingly, while surprisal shows a significant effect in first pass reading time, integration cost is significant only in regression path duration. This might point to a temporal disjunction with regard to working memory and prediction effects, however more work needs to be done in order to back this claim.

Recall that both surprisal and storage cost are motivated by predictive processing concerns. While surprisal captures the probability of a word given previous context, storage cost models the processing difficulty due to head prediction maintenance. Our results show that these two metrics might be capturing independent aspects of predictive processing. The correlation between storage cost and surprisal is marginal (r=-0.15). It is important to point out that so far there is no experimental support for storage cost in Hindi while there is support for surprisal. The reason for high storage cost in the Hindi eye-tracking data is varied, but it mostly happens in constructions with embedded structures. These embeddings include both verbal embeddings as well as complex noun phrases. There are some proposals that have argued for processing difficulty in English center-embeddings due to prediction maintenance [16] also see,[46]. Interestingly, surprisal shows up significant only in first pass reading time, while the storage cost seems to be a late emerging effect. The exact role of storage cost in Hindi sentence processing and its relation with surprisal will need further investigation.

## General Discussion

Our results are consistent with previous work on naturalistic reading in Hindi [21]. Results show the role of word-level predictors such as word frequency, word bigram frequency, word length, as well as sentence-level predictors such as storage cost, integration cost and surprisal. Building on previous work we demonstrated that both storage cost as well as surprisal are significant predictors of reading time. While surprisal shows up in an early measure, storage cost appears in a late measure. This could point to reflecting distinct predictive processes.

While the surprisal metric as computed by the transition-based parser was found to be a significant predictor of first pass reading time, we wanted to see if it could also account for some of the experimental data in Hindi. If some experimental data cannot be accounted by our automatically computed metric but can be theoretically explained by surprisal, then this will highlight the limitations of the parsing model that we employ. We discuss this next.

### Role of parsing model

Self-paced reading experiment data from Husain et al. [20] was used in order to test the prediction of the computed surprisal on the experimental data. In particular we use the Experiment 1 reading time data from their study. The experiment had a 2×2 design crossing relative clause type and verb distance from the relative pronoun. Examples 5 shows all the four conditions. The key manipulation was that the relative clause verb paD-hii/paDhaa thii ‘read’ was either ‘near’ or ‘distant’ from the relative pronoun jisne/jisko. In particular, the near condition although bringing the verb closer to the relative pronoun disrupted the default SOV word order in Hindi. For example, the object kitaab ‘book’ in Subject relative, Near (Non-canonical order) condition appears after the RC verb.

(5) a. Subject relative, Distant (Canonical order) vah laRkaa, / jisne / kitaab / that boy who ERG book bahut dilchaspii se / paDhii thii, / with much interest read had meraa dost / hai my friend is ‘That boy, who read the book with great interest, is my friend.

b. Subject relative, Near (Non-canonical order) vah laRkaa, / jisne / bahut dilchaspii se / that boy who ERG with much interest paDhii thii, / kitaab read had book meraa dost / hai my friend is ‘That boy, who read the book with great interest, is my friend.’

c. Object relative, Distant (Canonical order) vaha kitaab, / jisko / us laRke ne / that book which ACC that boy bahut dilchaspii se / paDhaa thaa / with much interest read had bahut moTii / hai very thick is ‘That book, which that boy read with great interest, is very thick’

d. Object relative, Near (Non-canonical order) vaha kitaab, / jisko / bahut dilchaspii se / that book which ACC with much interest paDhaa thaa / us laRke ne / read had that boy bahut moTii / hai very thick is ‘That book, which that boy read with great interest, is very thick’

**Figure 1:**
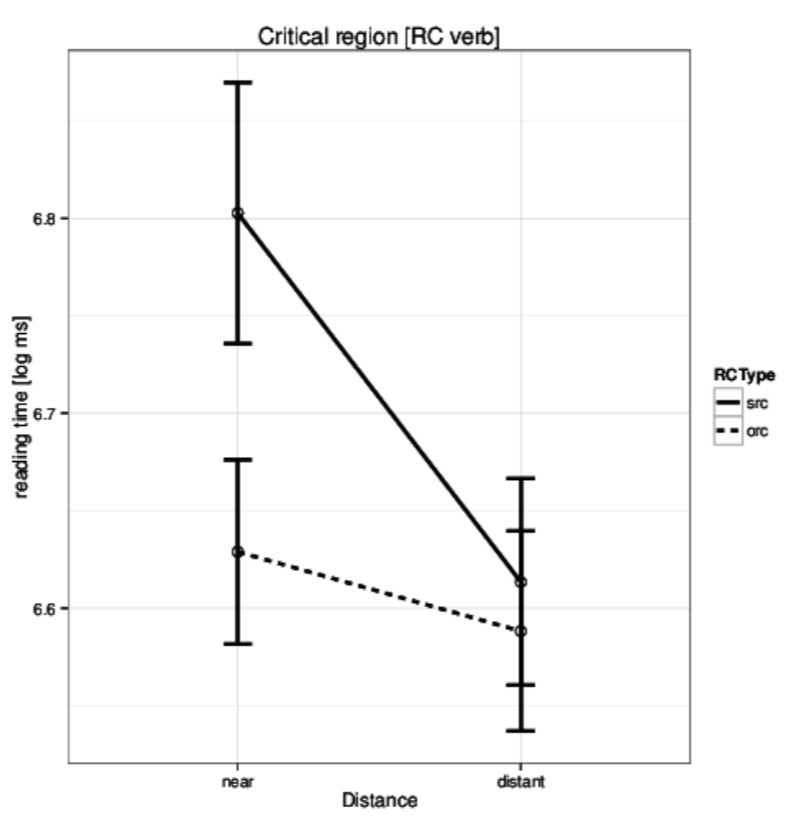
Husain et al. (2014) [20] Experiment 1: Reading times in log ms at the critical region (relative clause verb) for the four conditions.

One of the key results was that Hindi native speakers took longer to read the critical relative clause verb in the short condition. This can be seen in Figure 1. Surprisal can easily explain this pattern – in the subject relative clause the presence of Ergative case-marker on the relative pronoun predicts a transitive verb. Since the default word order in Hindi is SOV, an object is also expected to appear before the verb. In the ‘near’ condition the verb appears before the object thus negating this expectation. 9 The Hindi native speaker is therefore surprised to see the RC verb in this position leading to a higher reading time^5^.

As stated earlier, the ‘near’ conditions is expected to see a higher surprisal at the relative clause verb. It is therefore expected that the surprisal values computed by the parser should be higher in the near condition compared to distant condition. Surprisingly, we got the exact opposite results (t(23) = 4.6, p-value = 0.0001; mean of differences 0.14, 95% CI 0.08, 0.21). The t-test implied that surprisal, as calculated by us, does not account for the theoretical prediction of the surprisal metric in the case of these sentences. At the same time, the surprisal values computed by the parser have a significant effect on First-Pass Reading Time during naturalistic reading of the data discussed earlier. This shows that certain lexical/syntactic processes are being captured by the computed metric. One possible reason for this anomaly could be the nature of the parsing model that we use.

Two aspects of the parser model is worth highlighting here. First, transition-based models such as the one used in this study are known to take very local decision while ignoring the global sentential configuration [48]. This has been shown to adversely affect its performance in case of word order variability [17]. Previous work on modelling experimental data using surprisal have mainly used phrase structure parsers [18, 29]. These parsers assume a probabilistic phrase structure grammar (PCFG) that is induced from a treebank. The grammar rules in PCFG are directly associated with probabilities that are used to compute prefix probabilities. These prefix probabilities are then used to compute surprisal. These phrase structure rules (and therefore the associated parsing) can potentially capture the argument structure variability better compared to the dependency parsing using a transition-based system. Such an approach requires the availability of a phrase-structure treebank which is currently not available for Hindi.

The second aspect of the parser model relates to the feature set and labeled parsing. Our original feature set did not have the transitivity information of the verb. We tried adding transitivity information and more global features like the information about its first and second left-dependents but that led to reduction in parser accuracy. Further we could not add information about the dependency relation of the verb with its left-dependents since we were doing an unlabeled parsing. Perhaps a labeled parser might be able to capture this notion of surprisal. We intend to investigate this in future work.

So, while the automatically computed surprisal values do account for some variance in the eye movement data from naturalistic reading in Hindi, it is unable to correctly predict the experimental data discussed above. This shows that properties such as parser algorithm, feature set, grammar assumptions, etc. are critical for the predictive power of a parsing model. Investigating such properties will be critical in order to account for experimental data such as Kamide et al. [23]; R. P. Levy and Keller [30], etc. For example, Kamide et al. [23] argued that German native speakers are able to use the case-marking of the subject along with the selectional-restriction of the verb to predict the most appropriate object before its auditory onset. Similarly, R. P. Levy and Keller [30] have argued that introducing a dative case-marked noun phrase leads to facilitation at the verb in German. This is presumably because the dative case-marked noun phrase makes the prediction of the upcoming verb more precise.

Similar to our results Demberg and Keller [9] did not find an effect of integration cost in first pass reading time 6. M. F. Boston et al. [6], on the other hand used an alternative metric to integration cost – retrieval cost, and found it to be significant for all measures for higher values of parser parallelism. One reason for the differing results in these studies could be that retrieval cost captures working memory constraints over and above what integration cost captures. We discuss this issue next.

### Retrieval cost: An alternative to integration cost

Similar to the study by M. F. Boston et al. [6], we calculate retrieval based on the cue-based activation model [31]. The time taken to retrieve a chunk from the memory depends on its activation cost which is given as Equation 2:

**Figure:**
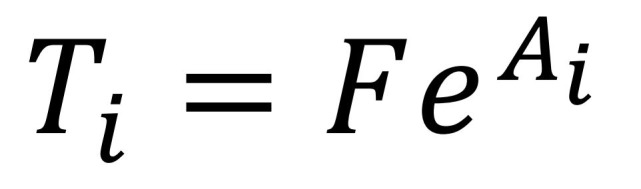


The activation of a memory chunk depends on two factors: decay and interference. This is shown in the following Equation 3:

**Figure:**
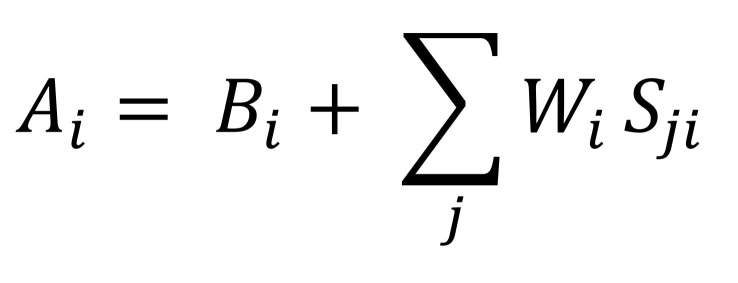


Here Bi is the decay term which ensures higher retrieval time if the word was last retrieved from the memory in the distant past. If *t_j__j=1_^n^* denote the set of times when the *i^th^* word was retrieved, Bi is given by Equation 4:

**Figure:**
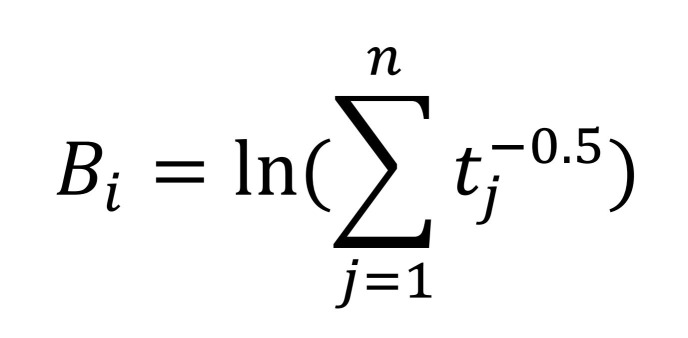


The interference term ensures that higher interference in retrieval (i.e. memory chunks with overlapping features) implies higher retrieval cost. It is computed as a weighted sum of Sjis which represent the strength of association.

**Figure:**
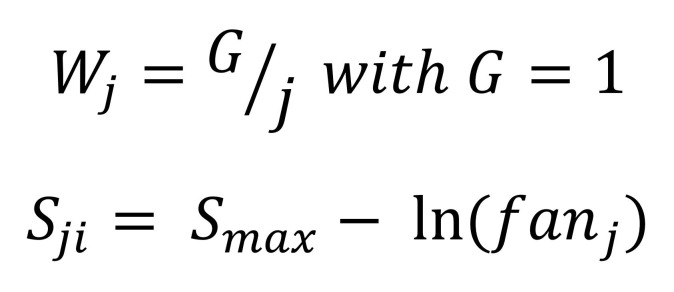


where fanj is the number of chunks that have the same feature as the jth retrieval cue. In our model, similar to M. F. Boston et al. [6], the part-of-speech category acts as a feature/cue and S_max_ is set to 1.5. Finally, productions in ACT-R are assumed to accrue a fixed cost of 50 ms and reading a cost of 1 ms to execute. Formation of a dependency arc accrues the cost of a retrieval along with two productions and a SHIFT operation accrues only one production cost.

While testing for the effect of retrieval, we leave out integration cost (IC) from the set of predictors since IC and retrieval are highly correlated (r=0.53). This is not surprising as both these measures formalize retrieval cost at the integration site. Also, like M. F. Boston et al. [6], we only consider points where the retrieval cost is non-zero and thus an effect of retrieval cost is expected.

The overall results are quite similar to those obtained earlier^7^. Interestingly, while retrieval cost is not significant for any of the three dependent measures for k=10; when the value of k is increased to 25, retrieval cost becomes marginally significant in the case of RPD (Table 8, see also Table 5, 6, 7). This seems to validate the results of M. F. Boston et al. [6] who also found significant effects of retrieval cost for higher parser parallelism. However, unlike them we did not find a significant effect of retrieval cost for all measures. The results without excluding points with zero retrieval cost are also very similar to the ones mentioned below, hence we skip them for brevity.

**Table 5:** Results of linear mixed-effects model on log first pass reading time

	Estimate(b)	Std. Error	t value
Intercept	5.501	0.023	237.72
Word complexity	0.002	0.003	0.67
Word frequency	6.750e-04	0.005	0.12
Word bigramfrequency	-0.013	0.003	-4.03
Syllable length	0.110	0.011	9.90
Storage cost	-9.006e-05	0.006	-0.01
Surprisal	0.016	0.004	3.75
Retrieval cost	-0.004	0.003	-1.14

**Table 6:** Results of linear mixed-effects model on log regression path duration.

	Estimate(b)	Std. Error	t value
Intercept	5.654	0.031	181.98
Word complexity	0.002	0.004	0.55
Word frequency	-0.004	0.007	-0.64
Word bigramfrequency	-0.023	0.003	-6.58
Syllable length	0.113	0.011	10.11
Storage cost	-0.015	0.007	-2.17
Surprisal	0.004	0.005	0.75
Retrieval cost	0.007	0.005	1.42

**Table 7:** Results of linear mixed-effects model on log total fixation time.

	Estimate(b)	Std. Error	t value
Intercept	5.618	0.030	182.30
Word complexity	0.004	0.002	1.68
Word frequency	-0.014	0.006	-2.09
Word bigramfrequency	-0.017	0.004	-4.26
Syllable length	0.129	0.010	11.85
Storage cost	0.016	0.006	2.46
Surprisal	0.011	0.004	2.33
Retrieval cost	-0.006	0.004	-1.52

**Table 8:** Results of linear mixed-effects model on log regression path duration (k=25)

	Estimate(b)	Std. Error	t value
Intercept	5.656	0.031	180.98
Word complexity	0.002	0.003	0.61
Word frequency	-0.006	0.007	-0.81
Word bigramFrequency	-0.024	0.003	-6.79
Syllable length	0.115	0.011	10.27
Storage cost	-0.014	0.007	-1.89
Surprisal	0.0001	0.005	0.03
Retrieval cost	0.009	0.004	1.91

How much (cross-linguistic) generalization can be drawn from our work and the eye-tracking corpus-based investigation in English and German? All these studies have found the effect of surprisal as well as memory costs on various eye movement measures. However, the exact measures for which these metrics are significant differ. For example, in this study we find the effect of surprisal only in first pass reading time, while M. F. Boston et al. [6] found the effect of (unlexicalized) surprisal for both early and late measures. In Demberg and Keller’s [9] study, the lexicalized surprisal does not show up in the results for first pass reading time. So, while there are some broad agreement between these results, because the modeling assumptions with respect to treebank annotations, parsing algorithm, nature of the predictors, parsing feature set, etc. are so varied, it is difficult to make any specific claims about cross-linguistics generalizations. A much more controlled modeling setup is needed in order to make any reasonable claim.

## Conclusion

In this work we used the Potsdam-Allahabad Hindi eye-tracking corpus to investigate the role of word-level and sentence-level factors during sentence comprehension in Hindi. We find that in addition to word-level predictors such as syllable length and uni- and bigram frequency, sentence level predictors such as storage cost, integration cost and surprisal significantly predict eye-tracking measures. Effect of retrieval cost (another working-memory measure) was only found for higher degrees of parser parallelism. Our work points to the possibility that surprisal and storage cost might be capturing different aspects of predictive processing. This needs to be investigated further through controlled experiments. Our study replicates previous findings that both prediction-based and memory-based metrics are required to account for processing patterns during sentence comprehension.

The results also show that model assumptions are critical in order to draw generalizations about the utility of a metric (e.g. surprisal) across various phenomena in a language.

## Acknowledgement

We would like to thank Marisa F. Boston and John Hale for clarifying certain doubts regarding the parser used in their paper [6]. We would like to thank Rajakrishnan Rajkumar for his comments and suggestions. We also thank Ashwini Vaidya for her comments on the work and for providing a resource for computing Hindi verb argument structure. Finally, we thank the two anonymous reviewers for their feedback.

## APPENDIX

In this section we discuss the technical details of the transition-based parser along with the data used in the study. We first discuss the data. Following this we list the feature specification file of the transition-based parser. Finally, we discuss the parser accuracy. The parser code and the eye-tracking data can be downloaded from: https://github.com/samarhusain/IncrementalParser

### Data

Dependency treebank. We used the sentences in the Hindi-Urdu treebank (HUTB) [4] to train our parser. The HUTB contains the dependency parse for around 12000 sentences along with morphological information (part-of-speech tag, category, lemma, case marker, chunk information, tense-aspect-modality and type of sentence) about each word in the treebank.

Eye-tracking corpus. We use eye-tracking data from the Potsdam-Allahabad Hindi Eye-tracking Corpus which contains different eye-tracking measures for 153 Hindi sentences. These sentences were selected from the HUTB treebank. The sentences were read by thirty graduate and undergraduate students of the University of Allahabad in the Devanagari script [21].

Feature Set: MERGE( InputColumn (POSTAG, Input [ 0 ] ) , InputColumn (POSTAG, Stack [ 0 ] ) , InputColumn (POSTAG, Stack [ 1 ] ) , InputColumn (POSTAG, Stack [ 2 ] ) ) MERGE( InputColumn (POSTAG, Input [ 0 ] ) , InputColumn (POSTAG, Stack [ 0 ] ) , InputColumn (POSTAG, Stack [ 1 ] ) ) MERGE( InputColumn (POSTAG, Input [ 0 ] ) , InputColumn (POSTAG, Stack [ 0 ] ) )

We have used a morphologically rich incremental feature set that includes the form, lemma, part-of-speech tag, category, tense-aspect-modality and case markers along with the chunking information of the top two elements of the stack and the top element of the buffer. We have not used the transitivity information of verbs and the gender, number and person of the words because they reduced the accuracy of the parser. The exact feature set used for the parser in the MaltParser format is given below:

Split ( InputColumn (FEATS_WITHOUT_GNP, Stack [ 0 ] ) , ‘|’ ) , Split ( InputColumn (FEATS_WITHOUT_GNP, Input [ 0 ] ) , ‘|’ ) , InputColumn (FORM, Stack [ 0 ] ) , InputColumn (FORM, Input [ 0 ] ) , InputColumn (POSTAG, Stack [ 0 ] ) , InputColumn (POSTAG, Input [ 0 ] ) , InputColumn (CHUNK ID, Stack [ 0 ] ) , InputColumn (CHUNK ID, Input [ 0 ] ) , InputColumn (POSTAG, Stack [ 1 ] ) , InputColumn (POSTAG, pred ( Stack [ 0 ] ) ) , InputColumn (POSTAG, head ( Stack [ 0 ] ) ) , InputColumn (POSTAG, ldep ( Input [ 0 ] ) ) , InputColumn (CPOSTAG, Stack [ 0 ] ) , InputColumn (CPOSTAG, Input [ 0 ] ) , InputColumn (CPOSTAG, ldep ( Input [ 0 ] ) ) , InputColumn (FORM, ldep ( Input [ 0 ] ) ) , InputColumn (LEMMA, Stack [ 0 ] ) , InputColumn (LEMMA, Input [ 0 ] ) , Merge ( InputColumn (CHUNK ID, Stack [ 0 ] ) , InputColumn (CHUNK ID, Input [ 0 ] ) ) , Merge ( InputColumn (CPOSTAG, Stack [ 0 ] ) , InputColumn (CPOSTAG, Input [ 0 ] ) ) , Merge ( InputColumn (POSTAG, Stack [ 0 ] ) , InputColumn (POSTAG, Input [ 0 ] ) )

We also tried our study with a simpler feature set which was used by Nivre [37]; M. Boston et al. [6]. The unlabeled accuracy for Hindi we obtained using this feature set was very low compared to what we get using the morphologically rich feature set. Also, the surprisal values we got using this feature set did not achieve a significant coefficient in any of the regression analyses. The details of this simplified feature set are given below:

### Parser Accuracy

Parser accuracy becomes critical in order to compute reliable surprisal values. The Unlabeled Attachment Score (UAS) for our parser is close to 88%. UAS is the proportion of words that are correctly attached to their parent. Using a simpler feature set [5] lead to lower accuracy (68%). UAS varies slightly with the value of k (which is the number of partial parses maintained in parallel), there is no clear increase in the accuracy as k increases. Surprisal values are computed using k = 10. This is done because the mean estimate of surprisal in the model (for FPRT) reaches maximum at k = 10.

The mean estimates and the standard deviations of the coefficient of surprisal in the linear mixed-effects regression for log(FPRT) for different values of k are given in the Table 9. As can be seen surprisal is significant for almost all values of k. Among the coefficients of surprisal in the case of First Pass Reading Time, we note that while the standard deviation of the estimate is nearly constant, the mean estimate first increases with k, reaches a maximum at k=10 and then starts decreasing again. Surprisal was not a significant predictor for both log(RPD) and log(TFT) for any value of k. We therefore do not show those figures here. For comparison we also show the retrieval cost figures (Table 10) at different values of k for regression path duration. We see here that retrieval cost reaches marginal significant for k=25, while it remains insignificant for lower k. For all other measures retrieval cost remains insignificant.

**Table 9:** Coefficient of surprisal for log first pass reading time for different values of k

k	Estimate(b)	Std. Error	t value
1	0.006	0.003	1.75
2	0.009	0.003	2.62
3	0.010	0.004	2.55
4	0.010	0.004	2.5
5	0.011	0.004	2.8
10	0.012	0.004	2.88
15	0.011	0.004	2.65
20	0.010	0.004	2.38
25	0.009	0.004	2.2

**Table 10:** Coefficient of retrieval cost for log regression path duration for different values of k

k	Estimate(b)	Std. Error	t value
2	0.010	0.006	1.75
3	0.010	0.005	1.79
4	0.008	0.005	1.50
5	0.007	0.005	1.41
10	0.007	0.005	1.42
15	0.007	0.005	1.40
20	0.007	0.004	1.59
25	0.009	0.004	1.91

## Footnotes

This information is part of the Husain et al. (2015) dataset [21].

version 3.1.2

version 1.17

Surprisal values are computed with a parser maintaining k=10 parallel parses. This k value was chosen as the significant effect of surprisal for first pass reading time was the highest (t=2.88) at this value (see Appendix for more details). For more details on parser parallelism and surprisal computation see, M. F. Boston et al. (2011) [6].

Husain et al. (2014)[20] also found a significant interaction effect, but this is not critical for the discussion here. Surprisal can also explain the interaction effect.

Actually, they found an effect but with a negative coefficient which is inconsistent with the claims of the dependency locality theory.

The significant effect of storage cost in Table 6 is inconsistent with the results discussed previously. We have no explanation for this effect.
